# Deletion of CD47 from Schwann cells and macrophages hastens myelin disruption/dismantling and scavenging in Schwann cells and augments myelin debris phagocytosis in macrophages

**DOI:** 10.1186/s12974-023-02929-0

**Published:** 2023-10-23

**Authors:** Miri Gitik, Gerard Elberg, Fanny Reichert, Michael Tal, Shlomo Rotshenker

**Affiliations:** 1 Medical Neurobiology, Faculty of Medicine, IMRIC, Hebrew University, Ein-Kerem Campus, 12272, 91120 Jerusalem, Israel; 2 Medical Neurobiology, Faculties of Medicine and Dentistry, Center for Research on Pain, Hebrew University, Jerusalem, Israel; 3 Present Address: Genomic Research Branch, Division of Neuroscience and Basic Behavioral Science (DNBBS), National Institute of Mental Health (NIMH), NIH, Rockville, USA

**Keywords:** CD47, SIRPα, Nerve injury, Wallerian degeneration, Macrophages, Microglia, Schwann cells, Phagocytosis, Myelin, Axon regeneration

## Abstract

**Background:**

Myelin that surrounds axons breaks in trauma and disease; e.g., peripheral nerve and spinal cord injuries (PNI and SCI) and multiple sclerosis (MS). Resulting myelin debris hinders repair if not effectively scavenged by Schwann cells and macrophages in PNI and by microglia in SCI and MS. We showed previously that myelin debris evades phagocytosis as CD47 on myelin ligates SIRPα (signal regulatory protein-α) on macrophages and microglia, triggering SIRPα to inhibit phagocytosis in phagocytes. Using PNI as a model, we tested the in vivo significance of SIRPα-dependent phagocytosis inhibition in SIRPα null mice, showing that SIRPα deletion leads to accelerated myelin debris clearance, axon regeneration and recovery of function from PNI. Herein, we tested how deletion of CD47, a SIRPα ligand and a cell surface receptor on Schwann cells and phagocytes, affects recovery from PNI.

**Methods:**

Using CD47 null (CD47−/−) and wild type mice, we studied myelin disruption/dismantling and debris clearance, axon regeneration and recovery of function from PNI.

**Results:**

As expected from CD47 on myelin acting as a SIRPα ligand that normally triggers SIRPα-dependent phagocytosis inhibition in phagocytes, myelin debris clearance, axon regeneration and function recovery were all faster in CD47−/− mice than in wild type mice. Unexpectedly compared with wild type mice, myelin debris clearance started sooner and CD47-deleted Schwann cells displayed enhanced disruption/dismantling and scavenging of myelin in CD47−/− mice. Furthermore, CD47-deleted macrophages from CD47−/− mice phagocytosed more myelin debris than CD47-expressing phagocytes from wild type mice.

**Conclusions:**

This study reveals two novel normally occurring CD47-dependent mechanisms that impede myelin debris clearance. First, CD47 expressed on Schwann cells inhibits myelin disruption/dismantling and debris scavenging in Schwann cells. Second, CD47 expressed on macrophages inhibits myelin debris phagocytosis in phagocytes. The two add to a third mechanism that we previously documented whereby CD47 on myelin ligates SIRPα on macrophages and microglia, triggering SIRPα-dependent phagocytosis inhibition in phagocytes. Thus, CD47 plays multiple inhibitory roles that combined impede myelin debris clearance, leading to delayed recovery from PNI. Similar inhibitory roles in microglia may hinder recovery from other pathologies in which repair depends on efficient phagocytosis (e.g., SCI and MS).

## Background

Myelin, a specialized extension of Schwann cells and oligodendrocytes in, respectively, peripheral and central nervous system (PNS and CNS), normally surrounds the larger diameter axons, enabling transfer and processing of information encoded in electrical signals. Myelin breaks in trauma (e.g., PNI and SCI) and disease (e.g., MS). Resulting myelin debris hinders repair if not rapidly and efficiently cleared. Potentially, Schwann cells and macrophages can clear myelin debris in PNS and microglia in CNS, Schwann cells through autophagy [1, 2] and phagocytosis [2–4], and macrophages and microglia through phagocytosis [4–7]. Regrettably, clearance is often deficient, leading to hindered repair.

We have been studying mechanisms that control myelin debris clearance using cultured primary macrophages and microglia in in vitro studies and PNI as a model in in vivo studies. PNI severs axons at lesion sites, leading to Wallerian degeneration distal to lesion sites [8]. In Wallerian degeneration, axons and myelin break, and mostly recruited macrophages and resident Schwann cells scavenge the debris; e.g., reviewed in [9, 10]. To regain function, severed axons must reach their denervated target cells by first crossing the lesion site, then entering and growing throughout the Wallerian degenerating nerve segment. The type of trauma determines if and how many of the severed axons successfully cross the lesion site. In crush injury, the nerve’s uninterrupted connective tissue enables efficient crossing. By contrast, the gap between proximal and distal nerve stumps that cut/avulsion injury forms obstructs crossing; e.g., discussed in detail regarding PNI in human [11, 12]. The nature of Wallerian degeneration that follows all types of nerve injuries determines whether regenerating axons that successfully crossed the lesion site will promptly reach their target cells by growing/regenerating throughout the distal nerve segment. Though possible, less than 50% of patients regain adequate sensory and motor functions [11, 12].

We focus in our studies on how Wallerian degeneration affects axon growth/regeneration. Observations in humans and studies in animal models suggest three factors that combined affect axon growth/regeneration. First is the length of the distal nerve segment that may vary from several millimeters to up to over one meter depending on species (e.g., mice versus humans) and site of trauma (e.g., near to versus distant from denervated target cells). Second is the decline with time of the capacity to support axon growth that initially develops in Wallerian degeneration [13, 14]. Third is the rate axons grow/regenerate. In humans, axon regeneration through Wallerian degenerating nerve segments was examined after avulsion injuries that required suturing of proximal and distal nerve stumps and after crush injuries that did not require surgical intervention. Sensory axons that regenerated initially 2.5 mm/day slowed to 0.5 mm/day and motor axons that initially regenerated 2 mm/day slowed to 1 mm/day [15, 16]. Thus, the initial slow rate of axon growth/regeneration slowed down further.

Leading causes of slow axon growth/regeneration in Wallerian degeneration are myelin debris (also referred to as degenerated myelin) and myelin-associated glycoprotein (MAG) [17–20]. Schwann cells and macrophages could alleviate axon growth inhibition by scavenging myelin debris, Schwann cells by autophagy [1, 2] and phagocytosis [2, 3], and macrophages by phagocytosis [4–7]. Yet, the rate of myelin debris removal is slower than the rate fast regenerating axons grow [21].

Previously, we showed that myelin debris inhibits its own phagocytosis in cultured primary macrophages and microglia through the binding of CD47 on myelin to immune inhibitory receptor SIRPα (also known as CD172a, SHPS-1, p84, gp93 and BIT) on the phagocytes, triggering SIRPα to generate “don’t eat” signaling [22, 23]. In this context, CD47 on myelin functions as a “don’t eat me” SIRPα ligand, as previously shown in other systems; e.g., [24, 25]. Next, we verified the in vivo significance of SIRPα-dependent inhibition of myelin debris phagocytosis using PNI as a model [26]. Macrophages from SIRPα−/− mice phagocytosed significantly more than macrophages from wild type mice, and furthermore, myelin debris clearance, axon regeneration and restoration of function were all faster in SIRPα−/− mice than in wild type mice [26].

We designed the current study to verify the in vivo significance of CD47 on myelin acting as a SIRPα ligand that triggers SIRPα-dependent phagocytosis inhibition in phagocytes [22, 23]. We used for this purpose CD47−/− mice that were subjected to broad behavioral testing, showing that CD47 deletion did not produce abnormalities in nociception nor in motor strength, coordination and locomotion, acts that require proficient functional integrity of sensory and motor peripheral nerve axons that depends, in turn, on proper myelination of the sensory and motor PNS axons [27]. In agreement with the notion that CD47 on myelin triggers SIRPα-dependent phagocytosis inhibition in phagocytes [22, 23] and our in vivo findings in SIRPα−/− mice [26], myelin debris clearance, axon regeneration and recovery of function were all faster in CD47−/− mice than in wild type mice. Unexpectedly, the onset of myelin debris clearance in CD47−/− mice preceded that in wild type mice, which was not the case in SIRPα−/− mice compared with wild type mice [26]. This discrepancy led us to look for roles other than acting as a “don’t eat me” SIRPα ligand through which CD47 may affect myelin debris scavenging. Indeed, CD47 (also known as IAP—integrin-associated protein) could play additional roles for two reasons. First, CD47 is a cell membrane receptor that regulates various functions by generating intracellular signaling (e.g., NO production, apoptosis and autophagy) and through lateral association with other cell surface receptors (e.g., integrins) [28–30]. Second, macrophages and Schwann cells express CD47 [22].

## Methods

### Animals

CD47 null (CD47−/−) and wild type mice colonies were housed at the Hebrew University Faculty of Medicine animal facility as previously reported [22]. Sex- and age-matched 8- to 12-week-old mice were used in experiments in accordance with the Israeli national research council guide for the care and use of laboratory animals and the approval of the Hebrew University institutional ethic committee.

### Surgical procedures

Surgery was performed under anesthesia on one hind limb of wild type and CD47−/− mice as we previously did [26]. Sciatic and saphenous nerves were exposed through small incisions in the overlaying skin. Freeze-crush injuries that enable axon regeneration were performed on saphenous nerves using a fine jeweler’s tweezer that was cooled in liquid nitrogen and then applied to nerves for 5 s, taking care to preserve the continuity of the epineurium. Avulsion injuries that do not enable axon regeneration were performed on sciatic nerves by removing a small nerve segment at mid-thigh level. Finally, the skin was sutured and sprayed with antiseptics.

### Assessment of the recovery of sensory function after nerve injury

We assessed recovery of sensory function as we previously did [26] using the flexion-withdrawal reflex, withdrawal of hind limbs in response to touching their paws with a blunt pin and von Frey monofilaments that produce punctate mechanical stimuli delivered mostly by Aδ axons; i.e., pinprick testing. Mice that had their saphenous nerve freeze-crushed were placed on an elevated wire mesh platform until calm, and then, testing of both injured and uninjured limbs was carried out by gently touching paws at areas that saphenous sensory axons normally innervate. Two investigators assessed the recovery of sensory function independently by testing all wild type and CD47−/− mice side by side at one-day intervals after surgery. Each mouse was tested for at least 3 days after function first returned to verify consistency.

### Isolation of primary thioglycollate-elicited peritoneal macrophages

Peritoneal cells were harvested in cold DMEM/F12 3 to 4 days after intraperitoneal injection of 1 mL of 3% thioglycollate (Difco, Detroit, MI, USA), as we previously did [31].

### Myelin isolation

The detailed protocol for isolating myelin was previously described [31]. Isolated myelin is “myelin debris” since intact myelin breaks during isolation.

### Phagocytosis of myelin debris

Phagocytosis was assayed as previously detailed [31, 32]. Macrophages were plated in 96-well tissue culture plates at a density that minimizes cell–cell contact in the presence of DMEM supplemented by 10% FCS. Non-adherent cells were washed out after 2 h and adherent cells left to rest overnight in DMEM supplemented by 0.1% delipidated BSA for experiments carried out in the absence of serum. Next, macrophages were washed in DMEM/F12 supplemented by 0.1% delipidated BSA, myelin debris added for 30 min and unphagocytosed myelin debris washed out thoroughly in DMEM/F12 supplemented by 0.1% delipidated BSA. At this time, the vast majority (over 95%) of the myelin debris is present in macrophages cytoplasm, thus phagocytosed, and only few adhered to the surface of macrophages. We showed this using immunofluorescence microscopy and a mAb against myelin-specific protein MBP (myelin basic protein) [31, 32]. Moreover, levels of visualized cytoplasmic (i.e., internalized/phagocytosed) myelin debris decreased with time after ending phagocytosis, indicating that macrophages degraded the internalized/phagocytosed myelin debris, MBP included. ELISA analysis of levels of cytoplasmic MBP/myelin debris (see below) revealed kinetics of phagocytosis and degradation similar to those shown by immunofluorescence microscopy [31, 32]. We further verified the validity of this assay by detecting 95% inhibition of myelin phagocytosis by cytochalasin-D [4, 33]. Using confocal microscopy, we also visualized live on going phagocytosis of myelin debris and using immunofluorescence confocal microscopy we visualized molecular changes in phagocyte cytoskeleton that control the initial steps and completion of phagosome formation on the surface of phagocytes [4, 33]. Notably, had we visualized at the early stages of phagosome formation just the myelin debris and not the cytoskeleton as well, we would have made the wrong interpretation of myelin debris attachment to the cell surface with no practical use to phagocytosis.

### Detecting and quantifying myelin debris phagocytosis by ELISA

This assay that was previously detailed [31] is based on the detection of the myelin-specific protein MBP in phagocytes. Since MBP is unique to myelin and macrophages do not produce it, MBP levels in phagocyte cytoplasm are proportional to levels of phagocytosed myelin debris. In brief, after exposure and then thoroughly washing out unphagocytosed debris (see above), phagocytes were immediately lysed (50 mM carbonate buffer, pH 10), lysates transferred to high protein absorbance plates (Thermo Fisher Scientific, Nunc International, USA) in equal volume of coating buffer (0.5 M carbonate buffer pH 9.6). Levels of MBP were determined by ELISA using rat anti-MBP mAb and matching control IgG (Bio-Rad Laboratories Inc., Hercules, USA).

When phagocytosis by macrophages from wild type mice was compared with phagocytosis by macrophages from CD47−/− mice, phagocytosis by each population was first normalized to the number of macrophages counted in 1-mm^2^ areas at the center of wells. Normalizing phagocytosis to cell number is required since phagocytes from the two mice strains may differ in their adherence properties, thus resulting in different number of adherent cells even when the same number of cells was initially seeded. To this end, phagocytes in replicate plates were fixed, stained and counted. Levels of MBP/phagocytosis in CD47−/− macrophages were calculated as percentage of MBP/phagocytosis levels in wild type macrophages normalized to 100%.

### Quantifying MBP content in nerve tissue

The detailed protocol used to quantify Galectin-3/MAC-2 [34] was previously adopted to quantify MBP in peripheral nerves [26, 35]. In brief, nerves were homogenized in 50 mM sodium carbonate buffer pH 9.0 supplemented with protease inhibitor cocktail (Sigma-Aldrich, Saint Louis, USA), protein concentration in cleared extracts was determined using the Bradford assay reagent (Bio-Rad Laboratories Inc., Hercules, USA) and adjusted to 5 µg/mL. Equal volumes (75 µL) of extracts and coating buffer (0.5 M carbonate buffer pH 9.6) were incubated overnight at 4 ℃ in 96-well high protein absorbance plates (Thermo Fisher Scientific, Nunc International, USA), and levels of MBP determined by ELISA using rat anti-mouse MBP mAb and matching control IgG (Bio-Rad Laboratories Inc., Hercules, USA).

### Immune fluorescence confocal microscopy

Nerves were cross-sectioned (8-μm) in a freezing microtome and sections blocked overnight (ON) in 10% FCS in PBS at 4 ℃. To visualize macrophages, sections were incubated ON at 4 ℃ in rat anti-mouse monoclonal antibodies (mAbs) M1/70 (Developmental Studies Hybridoma Bank, Iowa City, USA) and 5C6 (American Type Culture Collection, Rockville, USA) that were raised against the αM/CD11b subunit of complement receptor-3 (CR3) that mediates most myelin debris phagocytosis in phagocytes [4–6]. To visualize axons, sections were incubated ON at 4 ℃ in rat anti-neurofilament (anti-NF) IgG fraction (Sigma-Aldrich, Israel) diluted 1:5 in 10% FCS in PBS, washed in PBS, fixed in 4% neutral formalin in PBS for 20 min, washed in PBS, incubated for 40 min in FITC-conjugated rabbit anti-rat IgG (Jackson IR laboratories, PA, USA) (diluted 1:500 in 10% FCS in PBS), and finally washed in PBS. Microscopy was carried out in Olympus FluoView FV1000 confocal microscope.

### Electron microscopy

Tissues were fixed for 2 h in 2.5% glutaraldehyde/2% paraformaldehyde in 0.1 M Na cacodylate buffer, washed in 0.1 M Na cacodylate buffer, fixed for 1-h in 1% osmium/1.5% K-ferricyanide in 0.1 M Na cacodylate buffer, dehydrated in ethanol, and finally embedded in EPON (all were obtained from Electron Microscopy Sciences, USA). Thin sections were viewed using Tecnai-12 transmission electron microscope and photographed by CCD camera MegaView II and software AnalySIS 3.0.

### Statistical analysis

**Fig. 1:**
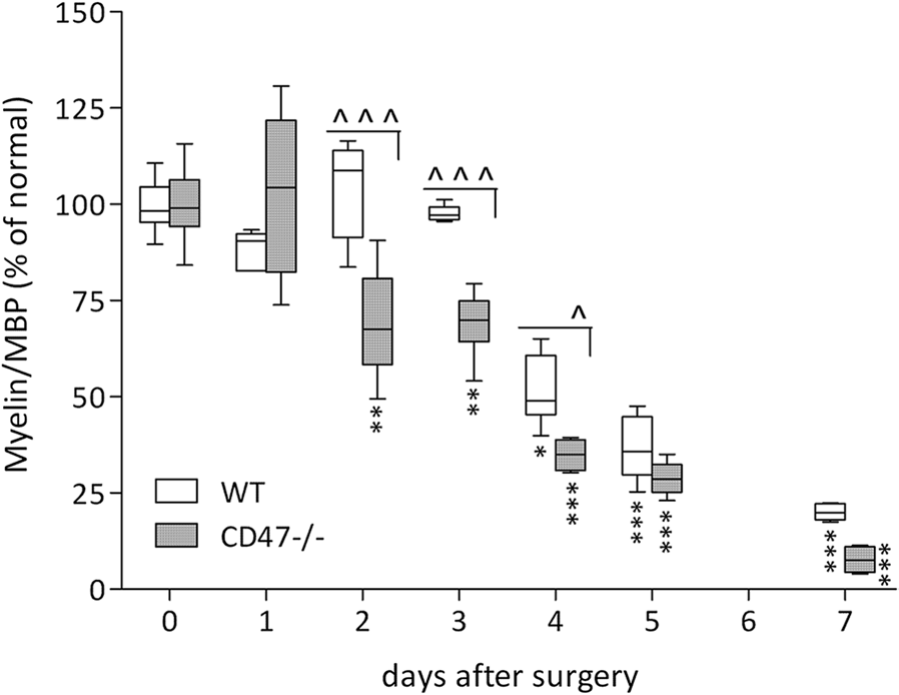
Earlier and faster clearance of myelin debris in CD47−/− mice than in wild type mice. Myelin-specific protein MBP levels were determined in intact nerves and in Wallerian degenerating sciatic nerve segments from wild type (WT) and CD47−**/**− mice at the indicated days after surgery. Levels of myelin/MBP are presented as percentage of levels in intact nerves (time 0) normalized to 100%. Box and whisker plot of myelin/MBP levels in 4 to 17 different nerves at the indicated days after surgery are given. Significance of difference of WT mice from SIRPα−**/**− mice at the indicated days after surgery is **^p** < 0.05 and **^^^p** < 0.001, by two-way ANOVA and the Bonferroni multiple comparisons posttest. Significance of difference between levels of myelin/MBP in intact nerves (day 0) and those at the indicated days after surgery is *p < 0.05, **p < 0.001 and ***p < 0.0001, by one-way ANOVA and the Dunnett posttest calculated for each mouse strain separately

The following statistical analyses were carried out using GraphPad Prism software: Gaussian distribution, the parametric unpaired *t* test and one- and two-way ANOVA, the nonparametric Mann–Whitney test, and the log-rank Mantel–Cox test. Data that passed the normality test were subjected to parametric statistics and those that were too small for testing for normality were subjected to nonparametric statistics. Quantitation of data in Figs. 1, 3B, 5, 6B and 7A & B is displayed in box and whisker plots; the box outlines the 25% to 75% range, the line in box represents the median, and whiskers extend to the highest and lowest values.

## Results

### *Myelin debris clearance starts sooner and is faster in CD47*−/−* mice than in wild type mice*

Nerve-tissue levels of the major myelin-specific protein myelin basic protein (MBP) are reduced in Wallerian degeneration as Schwann cells and macrophages scavenge and degrade myelin debris. We analyzed accordingly the timing of myelin debris clearance by determining the timing of the reduction in MBP levels in Wallerian degeneration in the absence of axon regeneration, as others and we did previously [2, 26, 35]. Of note, MBP contributes to myelin integrity and affects recovery from PNI; e.g., [36–39] (see Discussion).

Intact nerves from CD47−**/**− and wild type mice displayed similar MBP content. Compared with intact nerves, MBP content decreased significantly as of day 2 after surgery in CD47−**/**− mice but only as of day 4 after surgery in wild type mice. Overall, MBP content decreased significantly more in CD47−**/**− mice than in wild type mice on days 2, 3, and 4 after surgery. The advanced clearance of myelin in CD47−**/**− mice was also evident on days 5 and 7 after surgery though not statistically significant. Thus, significant clearance and degradation of myelin debris started sooner and continued faster in CD47−**/**− mice compared with wild type mice.

### *Sensory function recovers faster in CD47*−/−* mice than in wild type mice*

For studying how Wallerian degeneration affects the growth/regeneration of severed axons and thereby recovery of function from PNI it is advantageous to follow as many regenerating axons as possible. For that purpose, we inflicted freeze-crush injuries to sensory saphenous nerves. This type of injury severs all axons while preserving the continuity of the nerve connective tissue, enabling a large proportion of regenerating axons to cross the lesion site, then successfully enter the distal Wallerian degenerating nerve segment.

**Fig. 2:**
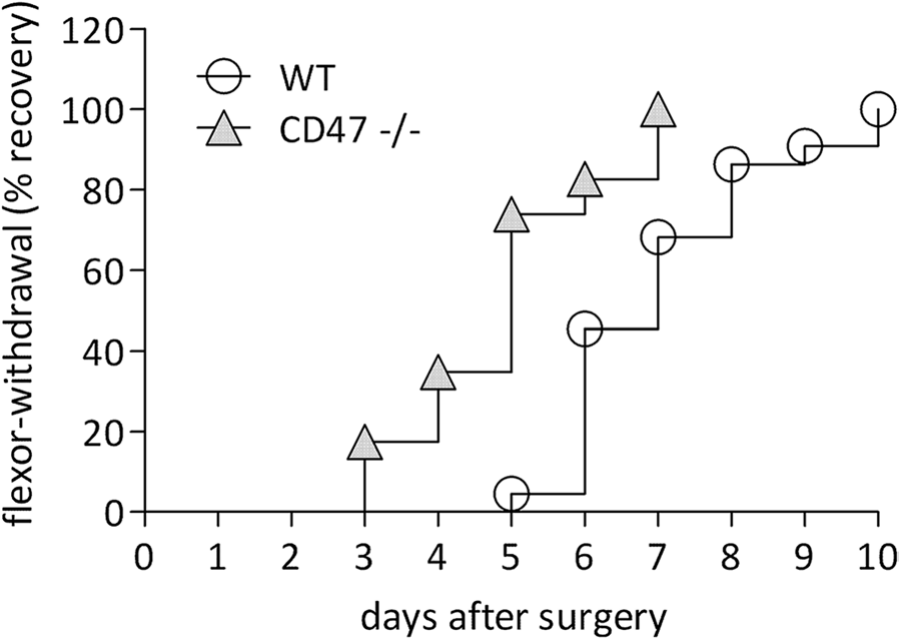
Sensory function recovers faster in CD47−/− mice than in wild type mice. The flexor-withdrawal reflex recovery curves display the cumulative percentage of CD47−/− and wild type (WT) mice that regained sensory function at each of the indicated days after surgery. Findings from male and female mice were combined since the two genders did not differ in recovery time. Overall, 22 WT mice and 23 CD47−**/**− mice were tested. Significance of difference of WT mice from CD47−/− mice is *p* < 0.0001, by the log-rank Mantel–Cox test

To test the recovery of sensory function, we used the flexor-withdrawal reflex, hind limb withdrawal in response to gently touching the paw. The saphenous and sciatic nerves provide sensory innervation to the hind limb paw and the sciatic nerve further supplies motor innervation to hind limb muscles. We freeze-crushed saphenous nerves at an average distance of 14 mm from paws. At the same time and same limb, we resected a segment of the sciatic nerve at mid-thigh level to prevent axon regeneration but spare hip joint flexion and thereby limb withdrawal. Hence, reflex recovery depended solely on successful regeneration and skin reinnervation by regenerating saphenous sensory axons. We operated on and tested wild type and CD47−**/**− mice side by side at 1-day intervals after surgery (Fig. 2). The reflex disappeared for at least 2 days after surgery, confirming successful sensory denervation of paws. In CD47−**/**− mice, the reflex returned in 17% of mice on day 3, median recovery was on day 5 and all mice had regained the reflex by day 7 after surgery. In wild type mice, the reflex returned in 4% of mice on day 5, median recovery was on day 7 and all mice regained the reflex by day 10 after surgery. Remarkably, on day 5 after surgery, 74% of CD47−**/**− mice regained the reflex, whereas only 4% of WT mice did so, reflecting 14.8-fold higher recovery rate in CD47−**/**− mice at that time. Thus, sensory function recovered significantly faster in CD47−**/**− mice than in wild type mice. Notably, unoperated CD47−**/**− and wild type mice responded equally to the stimuli that evoked the flexor-withdrawal reflex, in accord with a previous report that unoperated CD47−**/**− and wild type mice responded equally to nociceptive stimuli [27].

### *Severed axons regenerate faster in CD47*−/−* mice than in wild type mice*

**Fig. 3:**
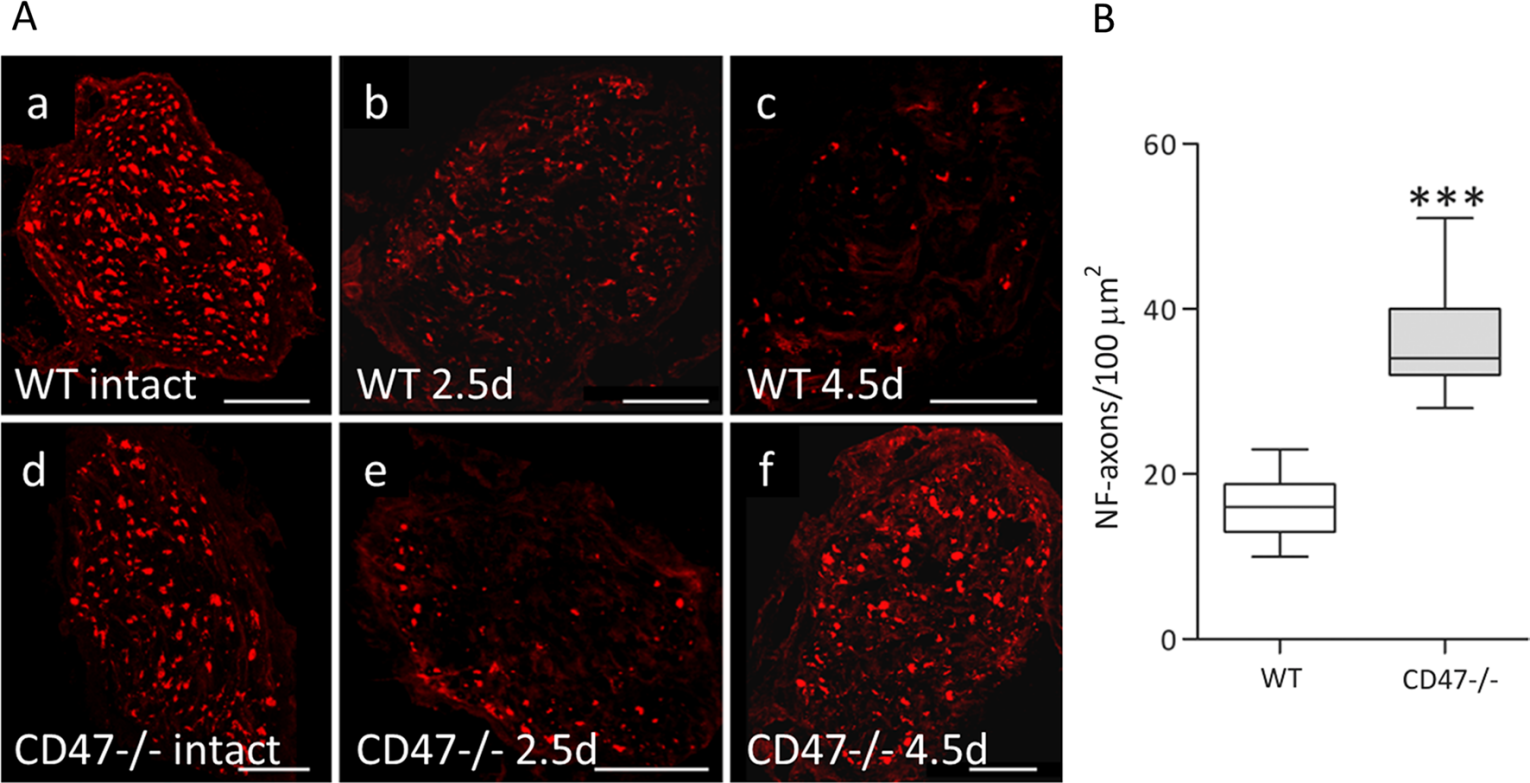
Severed axons regenerate faster in CD47−/− mice than in wild type mice. **A** Axons from wild type mice (WT; a, b and c) and from CD47−**/**− mice (d, e and f) were visualized in cryostat sections by immunofluorescence confocal microscopy using anti-neurofilament Abs (anti-NF; red). Intact and freeze-crushed saphenous nerves were sampled 10 to 12 mm distal to lesion sites at the indicated days after surgery. At 2.5 days (2.5 d) after surgery, NF immunoreactivity decreased in both WT mice (b) and CD47−**/**− mice (e). At 4.5 days (4.5 d) after surgery, NF immunoreactivity increased markedly more in CD47−/− mice (f) than in wild type mice (c). Bars: 50 μm. **B** Absolute counts of NF labeled axons (NF-axons) sampled 10 to 12 mm distal to lesion sites at 4.5 days after surgery (e.g. c and f). Box and whisker plot of NF-axons in 10 sections from 4 different WT mice nerves and 11 sections from 3 different CD47−**/**− mice nerves. Significance of difference between WT and CD47−**/**− mice is ***p < 0.0001, by unpaired t test

The earlier recovery of sensory function in CD47-**/**- than in wild type mice (Fig. 2) resulted most likely from faster growth/regeneration of their severed saphenous nerve sensory axons. To verify that this is the case, we visualized axons in intact and Wallerian degenerating saphenous nerves sampled 10 to 12 mm distal to lesion sites by positive immunoreactivity to neurofilaments (NF) (Fig. 3A). Of note, the reliability of NF immunoreactivity as a marker of regenerating PNS axons was verified by showing that comparable a number of regenerating axons were detected by both NF immunoreactivity and electron microscopy in the same cross-sections [40]. NF immunoreactivity decreased substantially in both CD47−**/**− and wild type mice at 2.5 days after surgery, indicating loss of axons due to rapid degeneration. NF immunoreactivity increased markedly in CD47−**/**− mice but less in wild type mice at 4.5 days after surgery, indicating quicker appearance of newly regenerating axons at the sampling site in CD47−**/**− mice than in wild type mice. Indeed, at 4.5 days after surgery, the number of NF positively marked axons was significantly 2.3-fold higher in CD47−**/**− than in wild type mice (Fig. 3B). These observations are in good agreement with the loss of sensory function in all mice for the first 2 days after surgery and functional recovery in 17% of CD47−**/**− mice but in none of wild type mice on day 3 after surgery (Fig. 2). Thus, sensory saphenous nerve axons grew/regenerated faster through Wallerian degenerating nerves in CD47−**/**− mice than in wild type mice.

### *Hastened and augmented *in vivo* disruption/dismantling and debris scavenging of myelin in CD47-deleted Schwann cells compared with CD47-expressing Schwann cells*

The clearance of myelin debris was faster in CD47−**/**− mice than in wild type mice, and furthermore, the onset of clearance in CD47−**/**− mice preceded that in wild type mice by 2 days (Fig. 1). We expected faster but not sooner onset of clearance based on the notion that deleting CD47 from myelin omits myelin’s CD47 role as a SIRPα ligand that normally triggers SIRPα-dependent phagocytosis inhibition in macrophages [22, 25]. Evidently, that was not the case. We searched, therefore, for other mechanisms through which CD47 may affect myelin debris scavenging. In this regard, CD47 deletion from Schwann cells and/or macrophages should be considered since the two cell types scavenge myelin debris in Wallerian degeneration and both express CD47 [22].

**Fig. 4:**
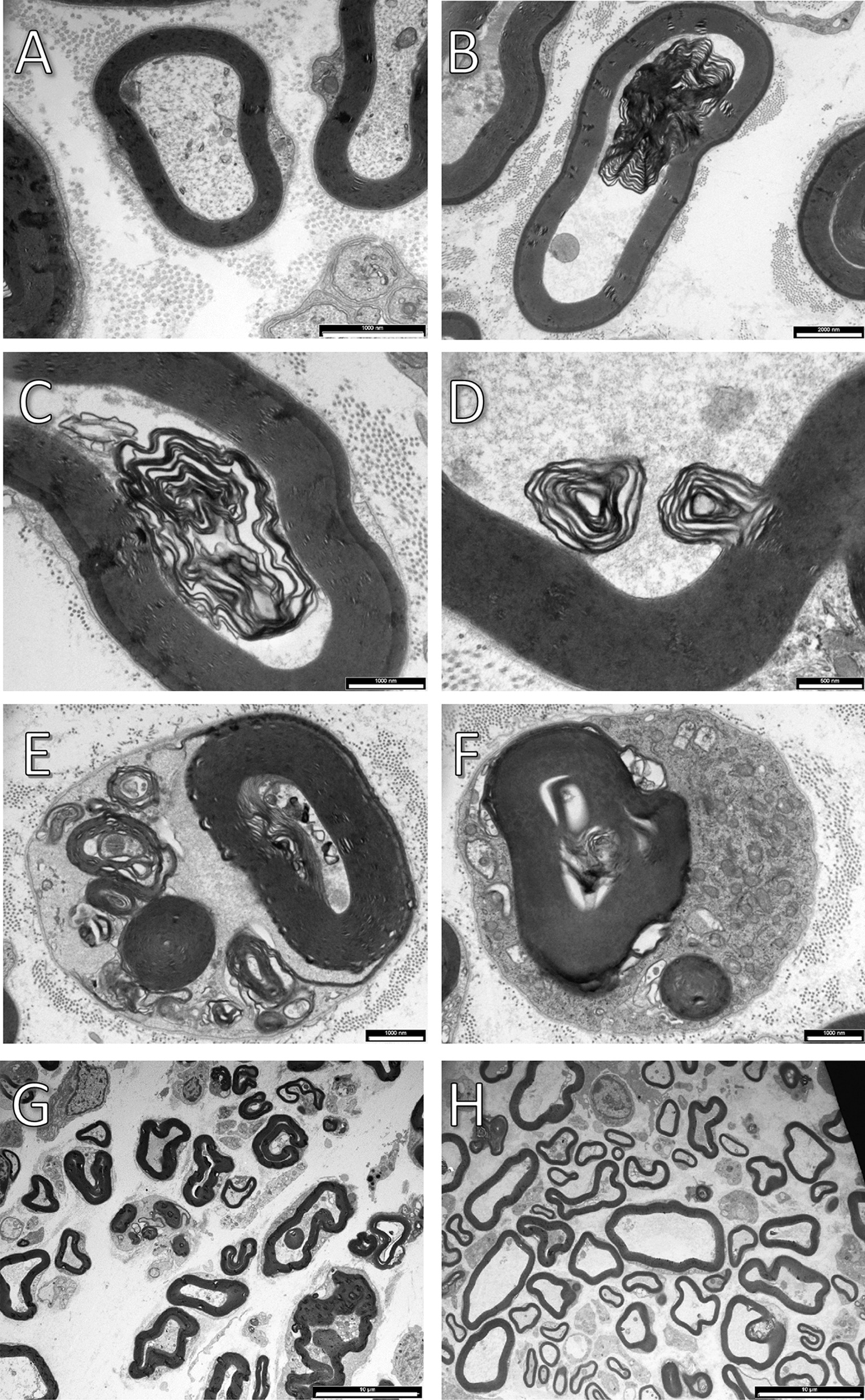
In vivo myelin disruption/dismantling and debris scavenging in CD47-deleted and wild type Schwann cells. Representative micrographs of cross sections from (**A**) intact and (**B**) through (**F**) Wallerian degenerating nerves taken 5 to 6 mm distal to but not including lesion sites on days 2 to 2.5 after surgery. **A** In CD47−**/**− mice, the flat myelin sheath forms tightly laminated spiral windings around intact axons. **B** through **F** In Wallerian degenerating nerves in CD47−**/**− mice, sections of tight laminated spiral windings loosen/unwind, exposing spaces between layers (**B**, **C** and **D**), forming small myelin coils of which some remain attached and some detach from large myelin spirals (**E**), and other become internalized into Schwann cells (**F**). Notably, microtubules and neurofilaments enrich the cytoplasm of intact axons (**A**), disrupted amorphous axonal cytoplasm embeds unwounded sections of myelin spirals (**B**, **C**, **D** and **E**), and numerous mitochondria enrich Schwann cells’ cytoplasm that embeds myelin debris (**F**). **G** and **H** Lower power micrographs of Wallerian degenerating nerves taken from (**G**) CD47−/− and (**H**) wild type mice. Bars: **A**, **C**,** E** and** F** 1 µm; **B** 2 µm; **D** 0.5 µm; **G** and **H** 10 µm

**Fig. 5:**
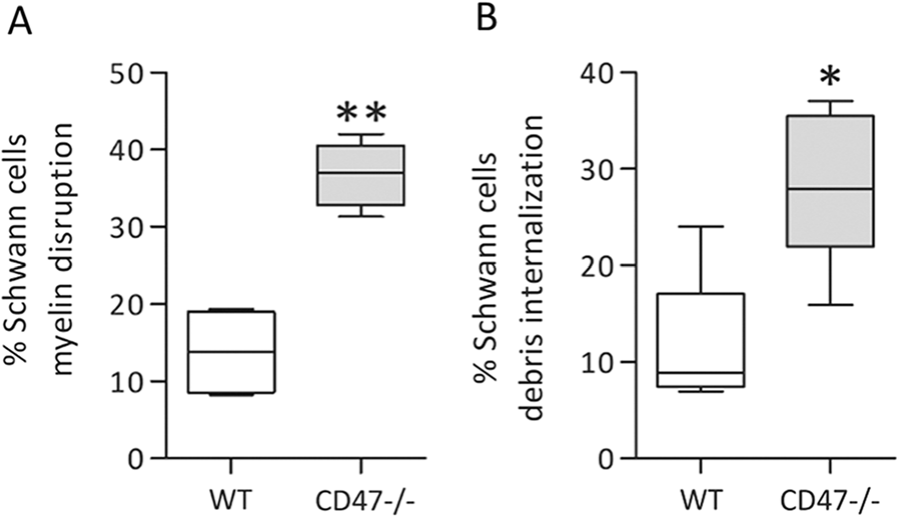
Myelin disruption/dismantling and scavenging in CD47-deleted Schwann cells exceed disruption/dismantling and scavenging in CD47-expressing Schwann cells. Schwann cells were randomly sampled in micrographs of cross sections of Wallerian degenerating nerves from wild type (WT) and CD47−/− mice taken on days 2 to 2.5 after surgery. Nerves were sampled 5 to 6 mm distal to but not including lesion sites (e.g., Fig. 4). Box and whisker plots of (**A**) percent of Schwann cells presenting disrupted myelin (e.g., Fig. 4B–D) and (**B**) percent of Schwann cells that contain myelin debris in their cytoplasm (e.g., Fig. 4F). In (**A**), total of 673 WT and 874 CD47−**/**− Schwann cells were sampled, respectively, in 4 WT and 6 CD47−/− injured nerves. In (**B**), total of 673 WT and 874 CD47−**/**− Schwann cells were sampled, respectively, in 5 WT and 6 CD47−**/**− injured nerves. Significance of difference between WT and CD47−**/**− mice is *p < 0.05 and **p < 0.01, by Mann–Whitney test

We focused first on Schwann cells, reasoning that disruption/dismantling of the normal tight multi-lamellar architecture of their myelin should precede myelin debris scavenging whether by Schwann cells or macrophages. If so, the expectation is that myelin disruption/dismantling will start sooner and/or be faster in CD47−**/**− mice than in wild type mice. To address this possibility, we studied myelin ultrastructure in Wallerian degenerating sciatic nerves in the absence of axon regeneration. We sampled injured nerves at a distance of 5 to 6 mm distal to but not including lesion sites on days 2 to 2.5 after surgery. This timing corresponds with clearance onset in CD47−**/**− mice but precedes clearance onset in wild type mice (Fig. 1). We observed a wide range of structural changes from normal in myelin and further detected myelin debris in Schwann cells’ cytoplasm in the two mice strains, but at higher frequencies in CD47−**/**− mice than in wild type mice (Figs. 4 and 5). As in wild type mice, the flat lamella of the myelin sheath coiled around intact axons forming tightly laminated spiral windings round them (Fig. 4A). In Wallerian degeneration, sections of the tightly laminated spiral windings loosened/unwound, exposing spaces between layers (Fig. 4B–D). Similar electron microscopic observations were previously made in experimental animals and human biopsies; e.g., [41–43]. Some of the unwound sections of myelin sheaths formed small coils of which some remained attached and some detached from the large spirals (Fig. 4E) and other became internalized into Schwann cells’ cytoplasm (Fig. 4F), as we showed previously [3]. Percent of Schwann cells that presented abnormal structure of their myelin (i.e., myelin disruption/dismantling) was significantly 2.7-fold higher in CD47−**/**− mice than in wild type mice (Fig. 5A) and percent of Schwann cells that contained myelin debris in their cytoplasm was significantly 2.4-fold higher in CD47−**/**− mice than in wild type mice (Fig. 5B). Thus, deletion of CD47 from Schwann cells hastened and augmented myelin disruption/dismantling and myelin debris scavenging in CD47−**/**− mice’s CD47-deleted Schwann cells, suggesting that CD47 that Schwann cells normally express impedes the disruption/dismantling of their myelin and Schwann cells’ ability to clear/scavenge myelin debris in Wallerian degeneration.

### *Comparable numbers of CR3-expressing phagocytes/macrophages in Wallerian degeneration in CD47*−/−* and wild type mice*

Recent studies suggest that recruited monocyte-derived macrophages outnumber resident macrophages during the first 7 days of Wallerian degeneration, and that mostly recruited macrophages clear myelin debris by phagocytosis [44–46]. Since macrophages normally express CD47 [22], deletion of CD47 from them could accelerate and increase their recruitment and/or augment their phagocytic capacity.

**Fig. 6:**
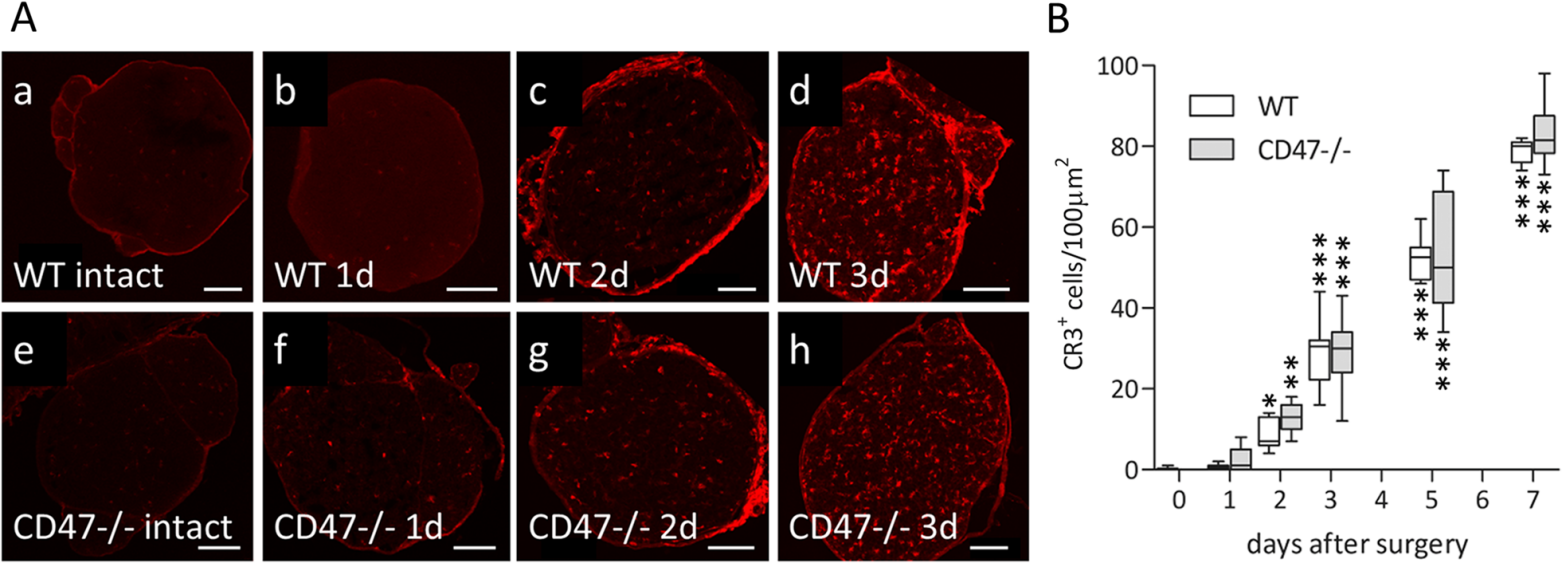
Comparable numbers of CR3-expressing cells/macrophages in Wallerian degeneration in CD47−/− and wild type mice. **A** Cells/macrophages were visualized in cryostat sections from Wallerian degenerating sensory saphenous nerves of wild type mice (WT; a, b, c and d) and CD47−**/**− mice (e, f, g and h) by immunofluorescence confocal microscopy using mAbs against αM/CD11b subunit of CR3 (red). Wallerian degenerating nerves were cross-sectioned 10 to 12 mm distal to lesion sites on days 1, 2 and 3 (1 d, 2 d and 3 d) after surgery. In intact nerves, CR3 immunoreactivity was barely detected in both WT mice (a) and CD47−/− mice (e). After injury, CR3 immunoreactivity increased progressively in both WT mice (b, c and d) and CD47−**/**− mice (f, g and h). Bars: 50 μm. **B** Absolute counts of CR3 labeled cells/macrophages (CR3^**+**^) in 100 µm^2^ of cross sections from intact (time 0) and Wallerian degenerating saphenous nerves (such as shown in A) at the indicated days after surgery. Box and whisker plots CR3^**+**^ expressing cells/macrophages in 6 to 18 sections from 4 different WT mice nerves and 4 different CD47−**/**− mice nerves that were sampled at the indicated days after surgery. Significance of difference from 0 days (intact) is *p < 0.05, **p < 0.01 and ***p < 0.0001, by one-way ANOVA and the Dunnett posttest, calculated for each mice strain separately

We addressed the issue of accelerated and increased recruitment by quantifying the number of cells that express CR3 (complement receptor-3; also known as MAC-1) that mediates much of the phagocytosis of myelin debris in macrophages and microglia, which we previously documented [5–7]. For this purpose, we sampled intact and Wallerian degenerating saphenous nerves 10 to 12 mm distal to lesion sites, visualizing CR3-expressing (CR3^**+**^**)** cells by detecting immunoreactivity to CD11b/αM subunit of CR3. CR3 immunoreactivity was infrequent and comparable in intact nerves in the two mice strains, which agrees with previously reported rare detection of 1.2 endogenous macrophages/100 μm^2^ in intact nerves [47], thus indicating that CD47 deletion did not change the number of endogenous macrophages. The number of CR3^**+**^ cells increased progressively to similar levels in the two mice strains from day 2 to day 7 after surgery (Fig. 6). This finding agrees with our previous observations that the number of cells expressing the macrophage specific F4/80 antigen increased progressively from 2.5 to 7 days after PNI [3] and with recent findings by others [44–46]. Noteworthy, CR3^**+**^ cells could be both macrophages and neutrophils [41]. However, most are macrophages since macrophages outnumber neutrophils through the entire period of myelin debris clearance (see Discussion). Taken altogether, the majority of CR3^**+**^ cells that we detected are most likely recruited monocyte-derived macrophages. The comparable number of CR3^**+**^ cells/macrophages in CD47−**/**− and wild type mice during the first 7 days of Wallerian degeneration suggests that it is unlikely that the earlier onset of myelin debris clearance (Fig. 1) resulted from differences in macrophage number between the two mice strains.

### *Augmented phagocytic capacity in CD47-deleted macrophages from CD47*−/−* mice compared with that in CD47-expressing macrophages from wild type mice*

**Fig. 7:**
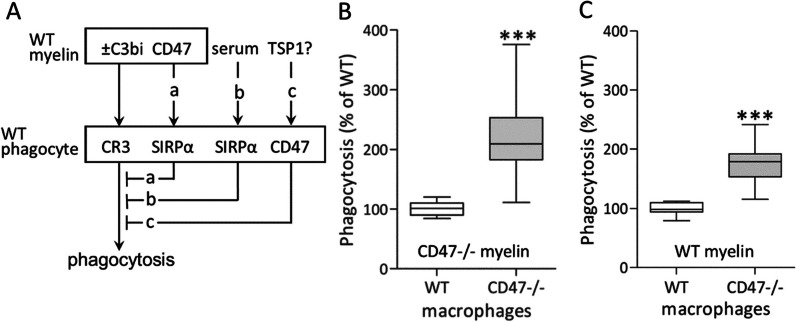
Greater phagocytic capacity in CD47-deleted than in CD47-expressing macrophages. **A** Activation (** →**) and inhibition (_**┴**_) of myelin debris phagocytosis—a schematic view. Wild type myelin (WT myelin) from wild type mice, either or not opsonized by complement protein C3bi (± C3bi), binds and activates CR3 that mediates much of the phagocytosis of myelin debris in context of traumatic injury [4–6]. CD47 on WT myelin (a) and serum (b) trigger each SIRPα-dependent phagocytosis inhibition in wild type macrophages (WT phagocytes). Tested hypothesis (c): CD47 on phagocytes triggers phagocytosis inhibition. A potential ligand that could activate CD47 is thrombospondin-1 (TSP1; see Discussion). **B** and **C** Phagocytosis in serum-free medium of myelin debris from (**B**) CD47−**/**− mice (CD47−**/**− myelin) and from (**C**) wild type mice (WT myelin) by WT and CD47−/− macrophages from WT and CD47−**/**− mice. In each experimental paradigm, phagocytosis levels in WT macrophages were normalized to 100% and phagocytosis levels in CD47−**/**− macrophages presented as percentage of phagocytosis in WT macrophages. Box and whisker plots of (**B**) 24 replicates in 7 experiments, (**C**) 18 replicated in 6 experiments. Significance of difference between WT and CD47−**/**− macrophages is ***p < 0.001, by unpaired *t* test. In CD47−/− macrophages, the 2.3-fold phagocytosis augmentation of CD47−/− myelin and the 1.7-fold phagocytosis augmentation of WT myelin differed one from the other significantly (*p* < 0.01, by unpaired *t* test; not marked in figure)

We showed previously that both CD47 and SIRPα are expressed on macrophages and microglia, whereas CD47 but not SIRPα is present on Schwann cells and myelin [21, 25]. These findings raise the possibility that deletion of CD47 from phagocytes could have altered their phagocytic capacity. We addressed this possibility by studying the phagocytosis of myelin debris from wild type and CD47−**/**− mice (WT and CD47−**/**− myelin) in cultured macrophages from wild type and CD47−**/**− mice (WT and CD47−**/**− macrophages) in the absence of serum (Fig. 7). Two experimental paradigms allowed us to test how deletion of CD47 from phagocytes affects their phagocytic capacity in the absence and presence of inhibition that CD47 on myelin normally induces in the absence of SIRPα-dependent phagocytosis inhibition that serum induces. We designed these experiments based on our previous findings in wild type phagocytes that CD47 on myelin and serum, each by their own and combined, triggered SIRPα-dependent phagocytosis inhibitions that were eliminated upon (a) removal of serum and deletion of CD47 from myelin and (b) upon deletion of SIRPα from macrophages (Fig. 7A, inhibitions “a” and “b”) and [22, 26]. In the first paradigm, CD47−**/**− macrophages phagocytosed significantly 2.3-fold more CD47−**/**− myelin debris than WT macrophages in the absence of serum (Fig. 7B). Here, the two genotypes phagocytosed CD47−**/**− myelin debris in the absence of SIRPα-dependent inhibitions that serum and CD47 on myelin normally induce. Thus, augmented phagocytosis in CD47−**/**− macrophages in the absence of SIRPα-dependent inhibitions resulted most likely from eliminating an inhibition that CD47 on macrophages normally generates (Fig. 7A, inhibitions “a”, “b” and “c” are not functioning). In the second paradigm, CD47−**/**− macrophages phagocytosed significantly 1.7-fold more WT myelin debris than WT macrophages in the absence of serum (Fig. 7C). Here, the two genotypes phagocytosed WT myelin debris in the presence of SIRPα-dependent inhibition that CD47 on myelin induces and the absence of SIRPα-dependent inhibition that serum induces. Thus, augmented phagocytosis in CD47−**/**− macrophages that took place in the presence of SIRPα-dependent inhibition that CD47 on myelin induces resulted most likely from eliminating an inhibition that CD47 on phagocytes normally generates (Fig. 7A, inhibition “a” is functioning and inhibitions “b” and “c” are not). Significantly, the 2.3-fold phagocytosis augmentation of CD47−**/**− myelin and the 1.7-fold phagocytosis augmentation of WT myelin differed one from the other significantly (*p* < 0.01, by unpaired *t* test). Thus, all findings together suggest that both CD47 and SIRPα that macrophages express inhibit phagocytosis and inhibitions by the two receptors are, at least in part, independent of one another and additive.

## Discussion

This study reveals two novel normally occurring CD47-dependent mechanisms that impede myelin debris clearance by acting as a cell surface receptor. First, CD47 on Schwann cells inhibits myelin disruption/dismantling and myelin debris scavenging in Schwann cells. Second, CD47 on macrophages inhibits myelin debris phagocytosis in macrophages. The two add to a third mechanism that we previously documented whereby acting as a SIRPα ligand, CD47 on myelin binds SIRPα on macrophages and microglia, triggering SIRPα-dependent phagocytosis inhibition in the phagocytes [22, 23, 26]. Thus, CD47 plays multiple inhibitory roles that combined impede myelin disruption/dismantling and debris clearance in injury-induced Wallerian degeneration. The resulting delayed clearance of myelin debris leads to slow axon growth/regeneration and protracted recovery of function. Similar CD47-dependent phagocytosis inhibition mechanisms may also contribute to protracted repair in other pathologies in which efficient phagocytosis is critical to repair (e.g., phagocytosis of myelin debris by microglia in MS and SCI, and phagocytosis of tumor cells by macrophages and microglia).

*The role of Schwann cells*. Myelin debris clearance in CD47−**/**− mice preceded that in wild type mice by 2 days (Fig. 1). We suggest that the earlier onset of myelin debris clearance in CD47−**/**− mice is mostly due to the elimination of a mechanism by which CD47 normally delays myelin disruption/dismantling and myelin debris scavenging in Schwann cells. We base this suggestion on our ultrastructural studies on days 2 to 2.5 after injury, a time window at which significant myelin debris scavenging had already begun in CD47−**/**− but not yet in wild type mice (Fig. 1). At that time, myelin disruption/dismantling and debris internalization into Schwann cells’ cytoplasm were already in progress and significantly greater in CD47−**/**− than in wild type mice, thus greater in CD47-deleted than in wild type CD47-expressing Schwann cells (Figs. 4 and 5).

The molecular mechanism by which CD47 delayed myelin disruption/dismantling and debris scavenging needs verification. We suggest nonetheless that it may relate, at least in part, to CD47’s established role as a cell surface receptor that inhibits autophagy [48] since Schwann cells scavenge myelin debris through autophagy [1, 2], albeit also by phagocytosis [2, 3]. Taken that CD47 is a receptor, deletion of CD47 from Schwann cells could have resulted in hastened myelin disruption/dismantling due to the loss of normally occurring signaling that CD47 generates in response to binding its cognate ligands SIRPα and/or TSP1 [28–30]. The loss of interaction between CD47 and TSP1 could have played a role. We base this suggestion on the following observations. First, TSP1 that is present at nodes of Ranvier in the endoneurium of intact PNS nerves persists in the endoneurium of injured PNS nerves [49, 50]. Second, at nodes of Ranvier, microvilli plasma membrane processes of myelinating Schwann cells are not associated with myelin, hence form direct molecular interactions with axons they wrap; e.g., [51]. Third, CD47 is expressed on plasma membranes of Schwann cells [22]. Taken all findings together, the structural architecture at nodes of Ranvier potentially enables extracellular TSP1 binding to CD47 on Schwann cells’ microvilli, triggering CD47 to generate down-stream signaling in Schwann cells’ cytoplasm. Moreover, as Wallerian degeneration proceeds, the continuous disruption/dismantling and scavenging of the myelin sheath exposes more of the myelinating Schwann cells’ plasma membrane, potentially broadening TSP1/CD47-dependent signaling. It is less likely that the loss of direct binding between CD47 and SIRPα could have played a role since such scenario requires the presence of SIRPα protein in intact PNS nerves, which our previous studies suggest is not the case. Using immunofluorescence microscopy, we detected SIRPα protein in macrophages recruited to Wallerian degeneration in vivo but did not detect SIRPα protein in intact PNS nerves nor in cultured explants of PNS nerves undergoing in vitro Wallerian degeneration in the absence of recruited macrophages and neutrophils [26]. Further, we detected SIRPα protein in cultured primary macrophages and microglia but not in cultured nerve-derived Schwann cells, myelin and fibroblasts [22]. Yet, we cannot rule out the possible presence of extremely low levels of SIRPα protein in intact peripheral nerves and cultured Schwann cells that immunofluorescence microscopy did not detect. Of note, SIRPα and CD47 mRNAs were detected in sorted Schwann cells using RNAseq without testing for the presence of SIRPα and CD47 proteins [52, 53]. We detected CD47 protein but not SIRPα protein in Schwann cells [22, 26]. The findings that SIRPα and CD47 mRNAs were detected whereas CD47 protein was but SIRPα protein was not, suggest that CD47 protein was translated and SIRPα protein was not. The occurrence of such scenarios is possible since translation does not always follow transcription; previously shown by others and us; e.g., [54, 55].

A point of consideration is whether genetic deletion of CD47 from myelin in CD47−**/**− mice could have affected the expression of other myelin molecule(s), which, in turn, could have contributed to accelerated disruption/dismantling of the myelin upon injury. Although we cannot rule out this possibility, the following warrants consideration. First, broad behavioral testing of CD47−**/**− and wild type mice revealed that the two mice strains did not differ in their response to nociceptive stimuli nor in motor strength, coordination and locomotion [27]. In line with these findings are our current observations that unoperated CD47−**/**− and wild type mice responded equally to stimuli that evoked the flexor-withdrawal reflex. All of the above mentioned actions depend on proficient functional integrity of myelinated sensory and motor PNS axons (i.e., fast propagation of information encoded in electrical signals, the action potential), which, in turn, depends on proper myelination of these axons (i.e., myelin thickness and length of the internodes and nodes of Ranvier). Therefore, the findings that CD47 deletion did not impair the functional integrity of myelinated peripheral axons suggest that deletion of CD47 did not impair myelination. Second, we detected comparable levels of MBP in intact PNS nerves in CD47−**/**− and wild type mice, suggesting that properties of myelin that MBP controls were not affected; e.g., [36–39] (see Discussion below).

*The role of CD47*−**/**−* macrophages*. We suggest that CD47-deleted macrophages in CD47−**/**− mice contributed little if any to the 2-day earlier onset of myelin debris clearance but could contribute significantly to faster clearance at later stages of Wallerian degeneration. We base this suggestion on the understanding that the phagocytic capacity of single macrophages and the total number of macrophages that are present in Wallerian degeneration at any given time together determine how much myelin debris a given population of macrophages clears. The phagocytic capacity of macrophages in CD47−**/**− mice exceeds that in wild type mice due to the deletion of CD47 from both macrophages and myelin that when expressed lead to phagocytosis inhibition (Fig. 7; discussed in detail in Results) and [22, 23, 26]. It is unlikely that this overall increase in macrophages’ phagocytic capacity could have contributed much to the 2-day earlier onset of myelin debris clearance in CD47−**/**− mice since only few macrophages were present during the first two days of Wallerian degeneration (Fig. 6). By contrast, it is most likely that the increased number of CD47-deleted macrophages at later stages of Wallerian degeneration (Fig. 6) enabled the entire growing population of CD47-deleted macrophages to fully implement their increased phagocytic capacity and so significantly contribute to faster clearance of myelin debris in CD47−**/**− mice.

We suggest two distinct CD47-dependent mechanisms that normally inhibit phagocytosis in macrophages and likely in microglia based on our previous and current findings. Previously we documented that CD47 expressed on myelin acted as a SIRPα ligand that triggered SIRPα to inhibit phagocytosis in macrophages and microglia [22, 23, 26]. Presently we suggest that CD47 inhibited phagocytosis in macrophages by acting as a cell surface receptor that macrophages express. We base this understanding on our current findings that deletion of CD47 from macrophages resulted in augmented phagocytosis (Fig. 7; we made similar observations in microglia; unpublished). The exact molecular mechanism by which CD47 on phagocytes could have inhibited phagocytosis needs verification. We suggest nonetheless that CD47 could inhibit phagocytosis, at least in part, by acting as cell surface receptor that upon activation lowers cAMP levels. We base this proposition on our previous findings that inhibiting cAMP signaling through PKA reduced myelin debris phagocytosis in macrophages and microglia [56] and findings by others that CD47 is Gi-coupled that upon activation reduces cAMP levels and subsequently signaling through PKA [57–59]. A potential ligand that could activate CD47 is TSP1 that macrophages and microglia among other cells produce and secrete [60, 61]. Our findings in this study that CD47 that macrophages express inhibited phagocytosis in both the presence and the absence of SIRPα-dependent phagocytosis inhibition (Fig. 7) further suggest that phagocytosis inhibitions by CD47 and SIRPα are, at least in part, independent of one another and additive.

Neutrophils infiltrate the Wallerian degenerating nerve early and transiently after PNI [41, 62]. The following scenarios warrant consideration in this regard. First, neutrophils, as macrophages, express both phagocytic receptor CR3 and CD47 [63]. Thus, it is highly probable, but needs verification, that deletion of CD47 affected phagocytosis in CD47-deleted neutrophils as it affected phagocytosis in CD47-deleted macrophages. Second, our findings that the timing and magnitude of the increase in the number of CR3-expressing cells in Wallerian degeneration in CD47−**/**− mice and wild type mice were comparable (Fig. 6) suggest that CD47 deletion did not affect the kinetics or magnitude of either neutrophils’ or macrophages’ recruitment in CD47−**/**− mice. Findings in other tissues support this understanding; compared to neutrophils and macrophages in wild type mice, deletion of CD47 from neutrophils and macrophages did not affect their recruitment to inflammatory peritonitis in CD47−**/**− mice, nor did CD47 deletion from neutrophils affect their recruitment to inflammatory peritonitis in wild type mice [63]. Further, the comparable number of CR3-expressing cells in intact PNS nerves in CD47−**/**− and wild type mice (Fig. 6) suggests that CD47 deletion did not affect the rare occurrence of endogenous macrophages. Third, it is most probable that the overall contribution of macrophages to the phagocytosis of myelin debris is considerably greater than that of neutrophils. This understanding is based on the observations that the presence of neutrophils in Wallerian degeneration was transient and further, that macrophages outnumbered neutrophils exceedingly through the entire period of myelin-debris removal; by 3 to1 on day 3 after PNI, and then, on day 7 after PNI, neutrophils were practically absent [41]. By contrast, the number of macrophages (i.e., cells expressing the murine macrophage specific F4/80 antigen) increased progressively during the same period [3, 62, 64, 65]. Fourth, targeted antibody-dependent deletion of neutrophils reduced myelin debris clearance in Wallerian degeneration in wild type mice (tested on day 7 after PNI), suggesting that neutrophils played a role in myelin debris clearance in Wallerian degeneration, but how was not tested, thus remained unknown [41]. We suggest that neutrophil depletion could have led to reduced clearance if neutrophils contributed to macrophage recruitment in Wallerian degeneration. We base this suggestion on findings in other tissues. Targeted antibody-dependent depletion of neutrophils resulted in impaired recruitment of macrophages to sites of injury, suggesting that neutrophils were instrumental in macrophage recruitment [66, 67]. Moreover, in acute inflammation, a more sustained presence of macrophages replaced an initial transient infiltration of short-lived neutrophils [68, 69]. The constitutive apoptotic death of the infiltrating neutrophils and the associated shedding of IL6R (interleukin-6 receptor) from their surface governed this transition, leading at the same to (a) IL6 trans-signaling that inhibited the production of leukocyte chemoattractants and (b) induced production of monocyte chemoattractants. In agreement, depletion of neutrophils resulted in reduced levels of soluble IL6R and reduced accumulation of macrophages in inflamed tissues [69]. We previously showed that fibroblasts produced significant levels of IL6 in Wallerian degeneration within hours after PNI and thereafter also macrophages contributed [70]. Together, the early production of IL6 [70] and early recruitment of neutrophils [41] in Wallerian degeneration suggest that neutrophils could have contributed to macrophage recruitment as they did in acute inflammation and injury in other tissues [68, 69].

*Recovery from PNI*. The faster removal of axon growth-inhibitory myelin debris in CD47−**/**− mice accounts most likely for faster axon growth/regeneration and so to facilitated recovery of function. Our current findings and observations by others suggest this. First, we showed in this study that the slower removal of myelin debris was associated with slower axon growth/regeneration and delayed recovery of function in wild type CD47-expressing mice compared with CD47−**/**− mice. Second, live in vivo imaging in wild type mice showed that myelin debris slowed axon growth/regeneration [21]. Third, the axon growth-inhibitory properties of myelin and MAG are well-documented [17–20]. Thus, CD47 normally prevents severed axons from fully implementing their regenerative potential by impeding myelin debris clearance through multiple mechanisms.

Interestingly, the major myelin-specific protein MBP plays a special role in recovery from PNI. We followed myelin clearance in this study by determining tissue levels of MBP in intact and Wallerian degenerating nerves. In doing so, we found that levels of MBP in intact nerves of CD47-/- and wild type mice were comparable, suggesting that CD47 deletion did not affect myelin properties and functions that MBP controls; e.g., myelin integrity and recovery from PNI. We base this understanding on the following studies. Studying MBP−**/**− and wild type mice, Smith-Slatas and Barbarese [38] showed that MBP levels were inversely proportional to the number of the Schmidt–Lanterman incisures (SLI), levels of connexin-32 and levels of MAG. Taken that SLI play an important role in the metabolic maintenance of the myelin [37, 71], MBP can affect myelin integrity by controling SLI number. Moreover, taken that MAG inhibits axonal growth/regeneration [17–20], MBP can affect recovery of function from PNI by controling MAG levels. Studying CD47−**/**− and wild type mice, Lutz et al., [39] showed that MBP, acting as a serine protease, cleaved the neural cell adhesion molecule L1, generating a 70-kDa L1 fragment that in culture facilitated neurite outgrowth and myelination by Schwann cells. In addition, the in vivo application of MBP to injured peripheral nerves resulted in increased levels of the 70-kDa L1 fragment, enhanced remyelination and facilitated functional recovery. Thus, MBP can affect recovery from PNI by controlling the levels of the 70-kDa L1 fragment. Martini et al. [36] suggested that the major components of the dense line of compact myelin MBP and P0 together determine myelin thickness. The authors based this understanding on their findings. First, mice deficient of both P0 and MBP (P0−**/**−/MBP−**/**−) were hypomyelinated and devoid of the major dense line. Second, myelin thickness was normal in (a) mice expressing P0 but not MBP (i.e., P0** + / + **/MBP−**/**−) and in (b) mice partially deficient of P0 and full expression of MBP (i.e., P0** ± **/MBP + **/** +). Third, myelin thickness was reduced in mice partially deficient of P0 and fully deficient of MBP (i.e., P0 ± /MBP−**/**−). All findings together led the authors to suggest, “When myelin forming Schwann cells are confronted with restricted resources, as, for instance, lack of MBP, myelin thickness strongly depends on the degree of P0 expression. On the other hand, MBP becomes a crucial determinant when only half of the dose of PO is available, since PO +  + /MBP– mice, but not PO + −/MBP– mice, form myelin of normal thickness.”

A point of consideration is whether genetic deletion of CD47 in CD47−**/**− mice could lead to accelerated axon growth/regeneration by affecting neurons directly. Observations made in human neuroblastoma cells and mouse primary cortical neurons show that transcription factor α-Pal/NRF-1 acting through CD47/IAP promoted neurite outgrowth and reduced expression of α-Pal/NRF-1 acting through CD47/IAP impaired neurite outgrowth [72]. Moreover, findings in cultured hippocampal neurons from CD47−**/**− and wild type mice showed that CD47 expression promoted and CD47 deletion impaired neurite outgrowth [73]. Thus, it is unlikely that genetic deletion of CD47 from neurons contributed to accelerated axon growth/regeneration in our current study.

## Conclusions

CD47 plays three inhibitory roles that combined impede myelin debris clearance in Wallerian degeneration, leading to slow axon growth/regeneration and retarded recovery from PNI. First, CD47 expressed on Schwann cells inhibits myelin disruption/dismantling and scavenging in Schwann cells. Second, CD47 expressed on macrophages inhibits phagocytosis in the phagocytes. Third, CD47 on myelin ligates SIRPα on macrophages, triggering SIRPα to inhibit phagocytosis in the phagocytes. It is highly likely that similar mechanisms may also hinder repair in other neurodegenerative pathologies in which myelin breaks. For example, phagocytosis inhibitions through CD47 that phagocytes express and through CD47 on myelin ligating SIRPα on phagocytes may both contribute to delayed myelin debris clearance in MS. Furthermore, these inhibitory mechanisms may impede phagocytosis in wild type phagocytes that express SIRPα and CD47 of any cellular target on which CD47 is expressed (e.g., red blood cells [24], platelets [74] and tumor cells [75, 76]).

## Data Availability

Not applicable.

## Abbreviations

SIRPα: Signal regulatory protein-α
MAG: Myelin-associated glycoprotein
MCSF: Colony stimulating factors
MBP: Myelin basic protein
CR3: Complement receptor-3
NF: Neurofilaments
PNS: Peripheral nervous system
CNS: Central nervous system
PNI: Peripheral nerve injury
SCI: Spinal cord injury
MS: Multiple sclerosis
IL6R: Interleukin-6 receptor
SLI: Schmidt–Lanterman incisures
